# A Single-Dose PLGA Encapsulated Protective Antigen Domain 4 Nanoformulation Protects Mice against *Bacillus anthracis* Spore Challenge

**DOI:** 10.1371/journal.pone.0061885

**Published:** 2013-04-29

**Authors:** Manish Manish, Amit Rahi, Manpreet Kaur, Rakesh Bhatnagar, Samer Singh

**Affiliations:** 1 Special Centre for Nano Sciences, Jawaharlal Nehru University, New Delhi, India; 2 Laboratory of Molecular Biology and Genetic Engineering, School of Biotechnology, Jawaharlal Nehru University, New Delhi, India; The Ohio State University, United States of America

## Abstract

*Bacillus anthracis,* the etiological agent of anthrax, is a major bioterror agent. Vaccination is the most effective prophylactic measure available against anthrax. Currently available anthrax vaccines have issues of the multiple booster dose requirement, adjuvant-associated side effects and stability. Use of biocompatible and biodegradable nanoparticles to deliver the antigens to immune cells could solve the issues associated with anthrax vaccines. We hypothesized that the delivery of a stable immunogenic domain 4 of protective antigen (PAD4) of *Bacillus anthracis* encapsulated in a poly (lactide-co-glycolide) (PLGA) - an FDA approved biocompatible and biodegradable material, may alleviate the problems of booster dose, adjuvant toxicity and stability associated with anthrax vaccines. We made a PLGA based protective antigen domain 4 nanoparticle (PAD4-NP) formulation using water/oil/water solvent evaporation method. Nanoparticles were characterized for antigen content, morphology, size, polydispersity and zeta potential. The immune correlates and protective efficacy of the nanoparticle formulation was evaluated in Swiss Webster outbred mice. Mice were immunized with single dose of PAD4-NP or recombinant PAD4. The PAD4-NP elicited a robust IgG response with mixed IgG1 and IgG2a subtypes, whereas the control PAD4 immunized mice elicited low IgG response with predominant IgG1 subtype. The PAD4-NP generated mixed Th1/Th2 response, whereas PAD4 elicited predominantly Th2 response. When we compared the efficacy of this single-dose vaccine nanoformulation PAD4-NP with that of the recombinant PAD4 in providing protective immunity against a lethal challenge with *Bacillus anthracis* spores, the median survival of PAD4-NP immunized mice was 6 days as compared to 1 day for PAD4 immunized mice (p<0.001). Thus, we demonstrate, for the first time, the possibility of the development of a single-dose and adjuvant-free protective antigen based anthrax vaccine in the form of PAD4-NP. Further work in this direction may produce a better and safer candidate anthrax vaccine.

## Introduction

Anthrax is primarily a disease of herbivores with occasional accidental human infection. It is caused by *Bacillus anthracis -* a Gram positive and spore forming bacterium. The ease of weaponization of *Bacillus anthracis* spores combined with the rapid course of the disease and the similarity of initial symptoms to common cold, make it a major biowarfare agent or bioterror threat. The mortality rate in inhalational anthrax is 45–90% even when the anthrax gets diagnosed early and followed by an aggressive antimicrobial schedule [1]. Furthermore, *Bacillus anthracis* spores can persist in the lung for 58 days; hence a prolonged antibiotic treatment is needed to prevent the disease relapse [2]. This scenario often makes the chemotherapy an ineffective measure for anthrax containment in case of a massive anthrax attack when supply of antibiotics could be limiting or when toxemia has already developed. Though there had been only limited casualties as a result of any anthrax outbreak in recent past, the anthrax spore attacks through postal mail in USA, 2001 [3] had exposed the limitations of the available vaccines in any emergency situation and prompted the research towards development of a more effective, safer and easily administrable vaccine [4]–[6]. Furthermore, the speculation that terrorist groups may have access to anthrax spores [7] or different rogue governments may use it as a biowarfare agent had kept the momentum of anthrax prevention research going.

The pathogenesis of *Bacillus anthracis* mainly depends on tripartite exotoxin protein complex and an anti-phagocytic poly-γ-d-glutamic acid capsule. Tripartite exotoxin is composed of protective antigen (PA), lethal factor (LF) and edema factor (EF). Protective antigen is the cell-binding moiety that acts as a carrier to translocate lethal factor and edema factor into the cytosol. Commensurate with the central role of PA in anthrax exotoxin activity, it is the major immunogen of all anthrax vaccines approved for human use [6], [8]. The commercially available anthrax vaccines for human use, *i.e.*, anthrax vaccine adsorbed (AVA) and anthrax vaccine precipitated (AVP), are made up of culture supernatant of toxigenic *Bacillus anthracis* adsorbed on alum or aluminum hydroxide [6], [8]. To generate and maintain effective immunity, 6 dose (3 subcutaneous doses at 2 week intervals followed by three more at 6, 12 and 18 months) of these vaccines are required along with an annual booster dose as long as the protection is needed (AVA; BIOTHRAX™ package insert). To alleviate concerns of batch to batch variation in antigen content, transient reactogenicity and the requirement of containment facility associated with AVA and AVP production [9]–[11], as expected from such culture supernatant based vaccines, the possibility of PA based anthrax vaccines have been extensively explored [6], [8]. However, the instability of PA remains a major concern in pharmaceutical formulation [12]–[14]. Immunization with PA alone induces poor protective response [15]. The problem of multiple booster doses is also not addressed by recombinant PA based vaccines. Furthermore, the recombinant protein based vaccines often require adjuvants to elicit a protective immune response. Though aluminum hydroxide or phosphate salts are the approved adjuvants in toxin based anthrax vaccines, it has been shown that aluminum hydroxide also degrades protective antigen on long-term storage [16]. Multiple endeavors are ongoing to develop a safer and more effective vaccine that may be more stable, non-reactogenic, require lesser number of doses, *etc*. [6], [8].

Biodegradable polymers offer a potential solution to many shortcomings of the current vaccines [17], [18]. The encapsulation of different immunogens in such polymer nanoparticles have been shown to provide stability and controlled sustained release, decreasing the need of boosters [17], [18]. Furthermore, as these biodegradable polymers have been shown to act as an effective adjuvant in terms of generating an immune response, a future vaccine could replace the current adjuvants, *i.e.*, aluminum hydroxide and aluminum phosphate, and hence their associated shortcomings in anthrax vaccine. As we wanted to develop a single-dose and adjuvant-free anthrax vaccine formulation, we chose to use poly (lactide-co-glycolide) (PLGA) as it has been extensively explored for pharmaceutical formulation [17]–[19]. PLGA is an FDA approved biodegradable polymer, that has been extensively tested and tried for the delivery of drugs, proteins, *etc.*, owing to its desirable physical properties and excellent biocompatibility, controlled release of antigens, targeted delivery potential and nontoxic degradation products [17]–[19]. Furthermore, the PLGA nanoparticle based vaccine formulations have been shown to improve the antigen uptake, presentation and cross priming by antigen presenting cells [17], [20]. The degradation rates of PLGA depend on the polymer and co-polymer ratio. PLGA often exhibit an initial burst release, followed by very slow release kinetics [17]–[19]. Thus, PLGA based nanoparticle vaccine could provide an alternative to the adjuvant use and eliminate the need of booster doses. The next challenge was to find a suitable immunogenic moiety that can both withstand the harsh condition of nanoparticle formulation and the low pH environment induced by degradation products of PLGA [17]. Inability of PA to withstand such harsh conditions had been proposed as a reason for lower immunogenicity of PA in a candidate vaccine that encapsulated PA in poly lactic acid (PLA) microspheres as compared to free PA [21]. A soybean oil based nanoemulsion encapsulating PA had been also tested as a candidate vaccine. It elicited Th1 response, unlike the desired Th1/Th2 response from anthrax vaccines [5]. Unlike complete PA molecule, the PA domain 4 (*i.e.,* PAD4) had been shown to withstand the low pH conditions and still maintain the structural integrity - similar to native PA, to bind with anthrax-toxin-binding cell-receptors [22]. The immunological properties of PAD4 had been also extensively studied [23]. The efficacy of PAD4 in generating a protective response against anthrax had been evaluated in conjunction with various formulations [6] including plant based expression system [24], alfalfa mosaic virus-mediated expression system [25], in combination with rabies virus glycoprotein as a carrier [26] and with influenza virus [27]. However, the potential of the domain 4 as an effective immunogen cannot be fully harnessed by employing whole PA molecule as the domain 4 is one of the most labile domains in the native PA molecule [28].

Considering these facts, we hypothesized that a PLGA encapsulated PAD4 nanoformulation could provide an effective and safer alternative to the currently available vaccines in terms of eliminating the need of adjuvant, requirement of booster doses and stability of immunogen in vaccine formulation. In the present work, we evaluated and demonstrated that PLGA can be successfully employed to encapsulate domain 4 of the protective antigen (*i.e.,* PAD4) without the loss of immunogen integrity. Furthermore, this nanoformulation comprising PLGA encapsulated PAD4 (*i.e.,* PAD4-NP) was able to generate protective immunity, comprised of both Th1 and Th2 response, against anthrax challenge without the need of any adjuvant or booster doses. The efficacy of this nanoformulation could be further improved upon to produce next generation of candidate anthrax vaccine.

## Materials and Methods

### General Reagents

Sterile deionized water was used for making all the buffers and aqueous phase preparations. Poly (D, L-lactide-co-glycolide), dichlormethane (DCM; Biotech grade 99.9% pure in Sure/Seal glass bottles), polyvinyl alcohol (PVA; 87–89% hydrolyzed: average M.W. 31,000–50,000), acetonitrile, and RBC lysis buffer were purchased from Sigma–Aldrich Corp. (Bangalore, India).

### Ethical Statement

All mice experiments were carried out as approved by Institutional Animal Ethics Committee, Jawaharlal Nehru University, New Delhi. Mice were housed in the individually ventilated animal caging system.

### Purification of Protective Antigen Domain 4 (PAD4)

The PAD4 was purified as described [24] with slight modifications. Briefly, PAD4 expression plasmid transformed *E. coli* cells were grown to O.D._600_ of 0.8 before being induced with 1 mM Isopropyl β-D-1-thiogalactopyranoside (IPTG). The cell culture was further allowed to grow for 4 h then pelleted down by centrifugation at 5000×g for 10 min. The bacterial cell pellet was lysed and solubilized in denaturing lysis buffer (8 M urea, 0.1 M phosphate buffer, 300 mM NaCl, pH 7.2) on a rotatory shaker for 2 h at a room temperature. Finally the insoluble fraction of cell lysate was removed by centrifugation at 15,000×g for 30 min. The supernatant was incubated with Ni-NTA slurry pre-equilibrated with denaturing lysis buffer on a rotatory shaker for 2 h. This mix was transferred to 5 ml propylene tube column and then slurry bound PA was renatured by passing a gradient of urea solution 8 M to 0 M (0.1 M phosphate buffer, 300 mM NaCl, pH 7.2). The steps after 4 M urea gradient were carried out at 4°C. The column was sequentially washed with 20 bed volume of 50 mM imidazole, 300 mM NaCl containing phosphate buffer (pH 7.4) and 10 bed volume of 100 mM imidazole, 300 mM NaCl containing phosphate buffer (pH 7.4). The column bound protein was eluted with 300 mM Imidazole and 150 mM NaCl containing 0.1 M phosphate buffer (pH 7.4) and concentrated using Macrosep® Advance Centrifugal Devices (3 kDa cutoff; Pall corporation, MI, USA).

### Preparation of PLGA Encapsulated PAD4 Nanoparticles (PAD4-NP)

The encapsulation of PAD4 protein in PLGA nanoparticles was carried out using w/o/w solvent evaporation method [20]. The PLGA (Sigma-Aldrich, Cat.# P2191–5G and lot # 051M1298V) used for PAD4 encapsulation had lactide and glycolide content in 50∶50 ratio (52∶48, as per the lot specification), inherent viscosity of 0.61 dL/g and its end groups were deactivated with lauryl alcohol. We dissolved 200 mg of PLGA in 4 ml of dichloromethane to make organic phase. A 100 µL aliquot of concentrated recombinant PAD4 (10 mg/mL) was used as internal aqueous phase for making PLGA encapsulated PAD4 nanoparticles. For preparing blank nanoparticles (Blank-NP), only phosphate buffered saline (PBS) was used as internal aqueous phase. First w/o emulsion was prepared by sonication of the PLGA and PAD4 mix using 2 mm stepped microtip at 35% amplitude for 60 seconds (750 W Sonic Vibra Cell Sonicator). The mix was kept on ice bath during whole sonication process. To increase the distance between emulsified droplets and minimize the aggregation of particle, 12 ml of external aqueous phase (diffused phase) containing 1% polyvinyl alcohol (PVA) was used. The w/o/w emulsion was prepared using 6 mm stepped tip at 30% amplitude for 110 sec. The emulsion was stirred for 6 h for the evaporation of DCM and hardening of the nanoparticles. Subsequently, PVA was removed by centrifugation at 15,000×g for 15 min and washed three times with sterile deionized water. The PLGA encapsulated PAD4 nanoparticles (PAD4-NP) were suspended in 5 ml of sterile deionized water and frozen in liquid nitrogen to avoid phase separation. The nanoparticles were kept at −80°C for 1 h then lyophilized at −54°C at 0.003 mbar for 18 h. Final PAD4-NP preparation was kept at −20°C for storage.

### Determination of PAD4 Encapsulation Efficiency and Quality

The encapsulation efficiency of PAD4 in PLGA nanoparticles was evaluated using micro-bicinchoninic acid assay (micro-BCA assay). Briefly, 10 mg of nanoparticles were suspended in 1 mL of acetonitrile, vortexed, centrifuged at 10,000×g for 10 min and the pellet was collected. The process was repeated three times and the final pellet was solubilized in 1% SDS. The protein encapsulation efficiency was estimated by BCA assay using bovine serum albumin dissolved in 1% SDS as standard. The purified PAD4 used for the encapsulation, and the encapsulated PAD4 recovered from PAD4-NP were analyzed by SDS-PAGE. The gels were stained with coomassie blue. The images were acquired by Molecular Imager GEL Doc XR System and analyzed using Quantity one software (Bio-Rad, Richmond, CA, USA). The overall nanoformulation process was also assessed in terms of yield of the nanoparticle formulation process {(total weight of dried encapsulated PLGA nanoparticles produced/total weight of PLGA polymer employed for making nanoparticles)×100%}, encapsulation efficiency {(weight of encapsulated protein/weight of the total protein used for encapsulation)×100%} and loading efficiency {(total weight of encapsulated protein/total dry weight of nanoparticles)×100%}.

### Morphological Characterization of Nanoparticles

Morphological characteristics of PAD4-NP were evaluated using scanning electron microscope Zeiss EVO40 (Carl Zeiss, Thronwood, NY*)* and transmission electron microscope JEM 2100F (Jeol Ltd., Tokyo, Japan). For scanning electron microscopy, dried PAD4-NP were spread on a carbon tape of an aluminum stub. The nanoparticles were made conductive by coating it with gold particles using a sputter coater (Polaron SC7640) at 2 KV for 200 second under inert Argon environment. The nanoparticles were viewed at electron high tension voltage of 20 KV and at 90.37 K X magnification. For the transmission electron microscope imaging, the nanoparticles were dissolved in deionized water at 0.1 mg/mL concentration, sonicated for 1 second, placed on carbon film with 200 mesh copper grids (Electron Microscopy Sciences, Hatfield, PA) and viewed under high vacuum, voltage of 200 KV and direct magnification of 25000X.

### Determination of Size, Dispersity, Zeta Potential and in vitro Release Profile of PAD4-NP

A diluted preparation of PAD4-NP (0.1 mg/mL) in PBS (pH 7.4) was used for the calculation of zeta potential and size. The zeta potential and particle size were calculated by measuring electrophoretic mobility and laser diffraction, respectively, on Nano ZS system (Malvern Instruments Ltd., Worcestershire, UK), employing a He-Ne laser (wavelength 633 nm). The polydispersity index (PdI) which indicates the variation in particle size was also measured. To study the release kinetics of PAD4 *in vitro*, the PAD4-NP aliquots suspended in 0.1 M PBS (pH 7.4) were incubated at 37°C. Supernatants were collected at different time points (4 h, 1 d, 3 d, 7 d, 14 d, and 28 d) and the protein content was determined using micro-BCA assay. The PAD4 release profile was generated by calculating the fractional protein or antigen release, *i.e.*, (PAD4 released/PAD4 encapsulated)×100%.

### Mice Immunization and Challenge with Anthrax Spores

For immunization studies, 8–10 week outbred female Swiss Webster mice procured from animal house, Jawaharlal Nehru University, were kept in a BSL-3 pathogen free environment during the course of the experiment. Mice were divided into four groups of 10 animals each. All groups were immunized with single dose, and the adjuvant-free immunization schedule was followed. The route of immunization was intraperitoneal (i.p.) and the dose volume used was 100 µL per injection. The animal groups were immunized with PAD4-NP (encapsulating 100 µg of PAD4), PAD4 (50 µg), blank nanoparticles (Blank-NP) or PBS alone. Sera from immunized mice were collected on day 14 and day 28 for the determination of antibody titers. For efficacy testing, the immunized mice (each group, n = 8) were challenged with a lethal dose of *Bacillus anthracis* Sterne strain (pXO1^+^pXO2^-^) spores (0.4×10^8^ spores per mouse) on day 40. The spores were prepared from a growing culture of Sterne strain of *Bacillus anthracis* as described previously [24].

### Determination of PAD4 Specific IgG isotype and IgG1/IgG2a Subtypes Titers

The enzyme linked immunosorbent assay (ELISA) was performed on a 96-well, flat bottom, polystyrene plates (Nunc-immuno™ maxisorp) to determine PAD4-specific IgG isotype and IgG1/IgG2a subtypes titers. The wells were coated overnight with 100 µL of PAD4 (5 µg/mL) diluted in PBS at 4°C. All wells were washed 3 times with PBST (PBS containing 0.05% tween-20), blocked with 10% FBS in PBS and then again washed five times with PBST using Tecan Columbus Pro microplate washer. Serial dilutions of antisera were made and added to the wells in triplicates and incubated for 2 h. The wells were washed five times with PBST, incubated with HRP conjugated goat anti-mouse IgG at 1∶10,000 dilution and its subtypes (IgG1 and IgG2a) at 1∶5,000 dilution for 1 h, washed five times with PBST, incubated with TMB substrate for 30 min. The reaction was stopped using 2N H_2_SO_4_ and the absorbance was measured at 450 nm using 570 nm as a reference wavelength (Tecan Sunrise microplate reader). The endpoint titer was calculated for each serial dilution with confidence interval 99% for increased specificity as described previously [29]. The endpoint titer was calculated as the reciprocal of the highest dilution having absorbance above the cutoff.

### Evaluation of Secreted Cytokines After in vitro Stimulation of Splenocytes

The spleens from two mice were harvested 40^th^ day post immunization. The splenocytes were spilled off from the spleen capsule slices using frosted slides. The red blood cells were lysed using RBC lysis buffer. The splenocytes were suspended in DMEM medium supplemented with 10% FBS and 50 µM mercaptoethanol. The splenocytes were plated at 8×10^4^ cells per well in 24 well tissue culture plates. The cells were stimulated with 1 µg/mL of ConA (positive control), 5 µg/mL of test sample or only medium (negative control). The culture supernatants were collected after 36 h of incubation and the cytokine levels were evaluated using Opt-EIA kit (BD Biosciences Pharmingen, San Diego, CA) as per manufacturer’s instructions.

### Statistics

The results are reported as mean ± SE. The antibody endpoint titer is reported as geometric mean. The statistical significance in antibody titer and cytokine level data was calculated by Student’s two-tailed t-test unless noted otherwise. The anthrax spore challenge experiments were evaluated using Kaplan-Meier survival estimates (GraphPad Prism, La Jolla, CA). The survival curve of PAD4 and PAD4-NP immunized mice were compared using Log-rank (Mantel-Cox) Test and Gehan-Breslow-Wilcoxon Test. Statistically significant differences between the groups are highlighted (* for P value between 0.01 to 0.05, ** for P values between 0.01 to 0.001, *** for P values <0.001).

## Results

**Figure 1:**
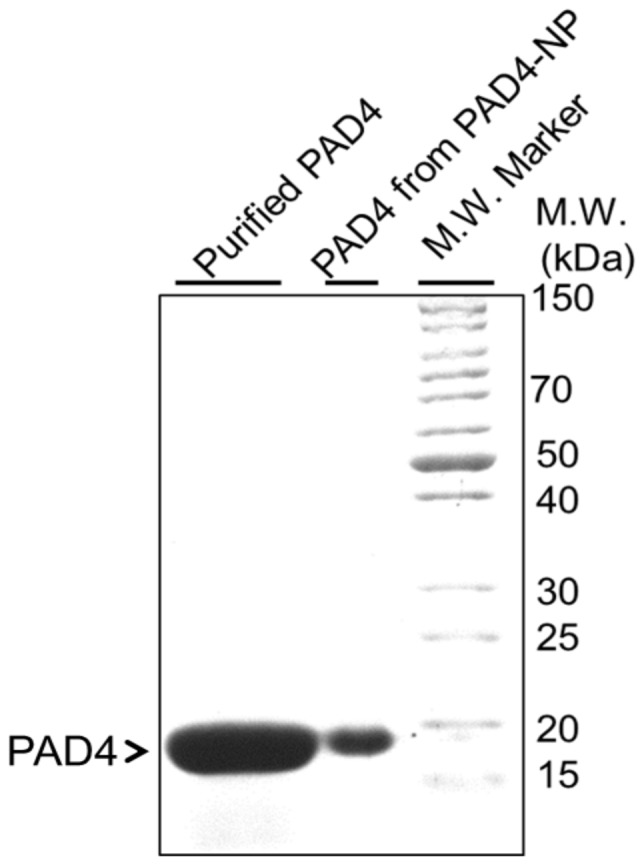
Purified protective antigen domain 4 (PAD4) can be successfully encapsulated in PLGA. The purified PAD4 from PAD4 expressing *E.coli* cells (Ni-NTA chromatography) that was used for making PAD4-NP by w/o/w method (left lane), PAD4 recovered from PAD4-NP (middle lane) and protein molecular weight ladder (right lane), were subjected to 10% SDS-PAGE followed by coomassie blue staining. Note the purified PAD4 (19 kDa, >90% pure) did not get degraded during nanoformulation process (compare middle lane with left lane).

The recombinant PAD4 purified from PAD4 expressing *E. coli* cells as described [24] **,** was further concentrated using Macrosep® Advance Centrifugal Devices (3 kDa cutoff) and the final protein preparation was analyzed by SDS-PAGE for PAD4 content and purity (Fig. 1, left lane). The protein preparation was found to be >90% pure. The concentration of protein was determined by micro-BCA assay. This purified concentrated PAD4 preparation was further used for encapsulation/loading in PLGA nanoparticles. The w/o/w solvent evaporation method was employed for making PAD4 containing PLGA nanoparticles, *i.e.*, only internal aqueous phase contained the PAD4 protein which was encapsulated inside a PLGA layer. The ratio of aqueous phase (containing PAD4) to oil phase (containing PLGA) was kept low to make nano-size particles as this ratio directly affects the particle size, *i.e.*, the greater the internal aqueous phase the bigger dimension particles would get made. The PAD4 containing PLGA nanoparticles (*i.e.,* PAD4-NP) were then characterized and evaluated for providing protective immunity against *Bacillus anthracis* spore challenge.

### Characteristics of PLGA Encapsulated PAD4 Nanoparticles

We used w/o/w solvent evaporation method for the preparation of the nanoparticles. The ratio between internal aqueous phase (containing PAD4) and continuous organic phase (containing PLGA) was 1∶40, whereas the ratio between organic phase and diffused phase (external aqueous phase) was kept 1∶4. The PLGA encapsulated PAD4 nanoparticles, PAD4-NP, were assayed for the antigen content and process yield. The dried nanoparticles were weighed and the yield of the process was estimated to be 73.12±2.37%. The nanoparticles were lysed using acetonitrile and the encapsulated antigen content was calculated using micro-BCA assay. The encapsulation efficiency of PAD4 nanoparticles was 73.62±3.19%. The loading efficiency of the nanoparticles was found to be 0.5±0.005%.

The molecular integrity of the PAD4 in the PAD4-NP formulation was also assessed to determine whether the antigen PAD4 withstood the shear force and organic solvents to which it was exposed during PLGA encapsulation process to make PAD4-NP. The PAD4 that was precipitated on dissolving PAD4-NP in acetonitrile was solubilized in Laemmli sample buffer and then analyzed by SDS-PAGE for integrity (Fig. 1, middle lane). A single band of ∼19 kDa (calculated M.W. 19.4 kDa) was observed after coomassie blue staining of the gel. It indicated that even under the harsh condition of nanoparticle formulation, PAD4 remained structurally intact and did not get degraded during the process (Fig. 1, compare recovered PAD4 in middle lane with PAD4 used for making PAD4-NP in left lane ).

**Figure 2:**
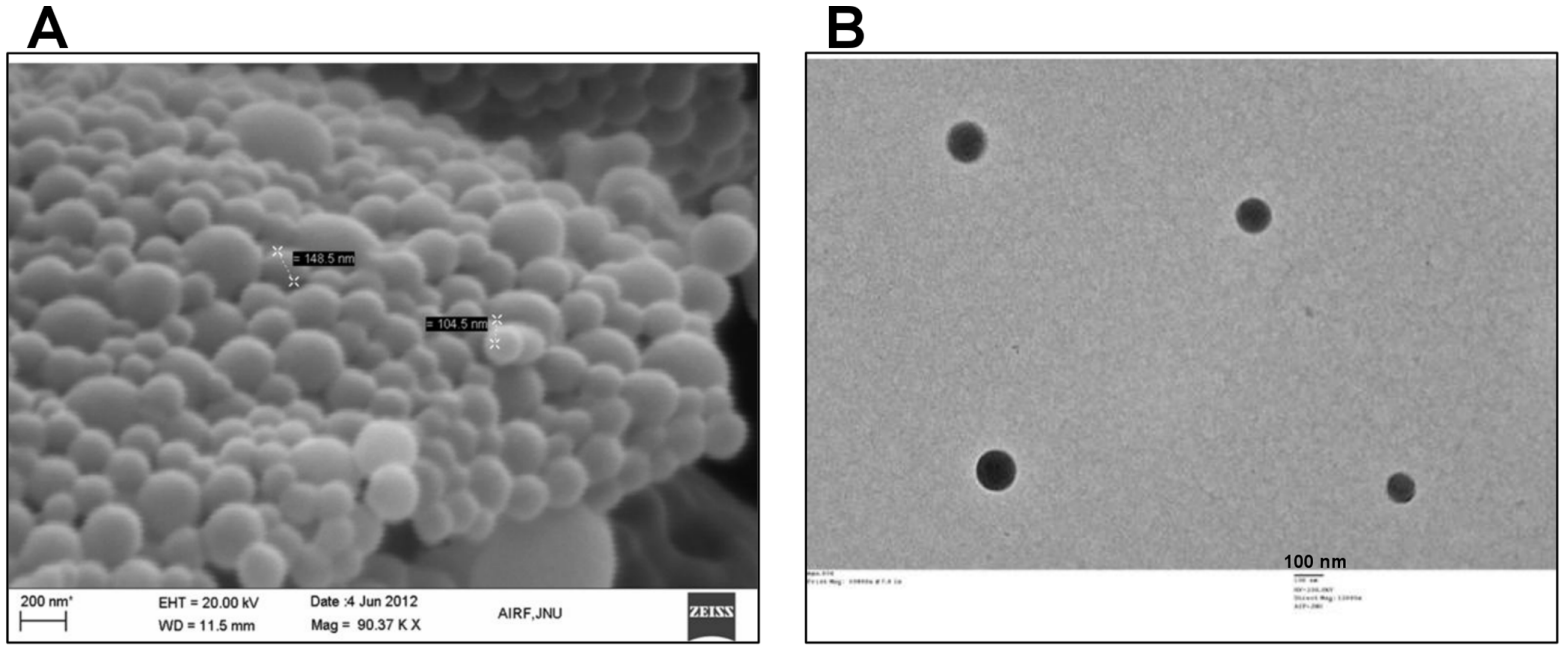
Surface morphology of PAD4-NP. The PLGA encapsulated PAD4 nanoparticles were visualized for surface morphology: (**A**) A scanning electron microscopy image (scale bar is 200 nm) (**B**) A transmission electron microscopy image (scale bar is 100 nm).

A smooth spherical nanoparticle formulation is considered the best as an antigen depot (adjuvant) and to create a controlled release formulation that could eliminate the need of booster doses in vaccination [17]–[19]. We visualized the PAD4-NP using scanning electron microscope (Fig. 2A) and transmission electron microscope (Fig. 2B). The nanoparticles made were smooth surfaced and spherical in shape.

**Figure 3:**
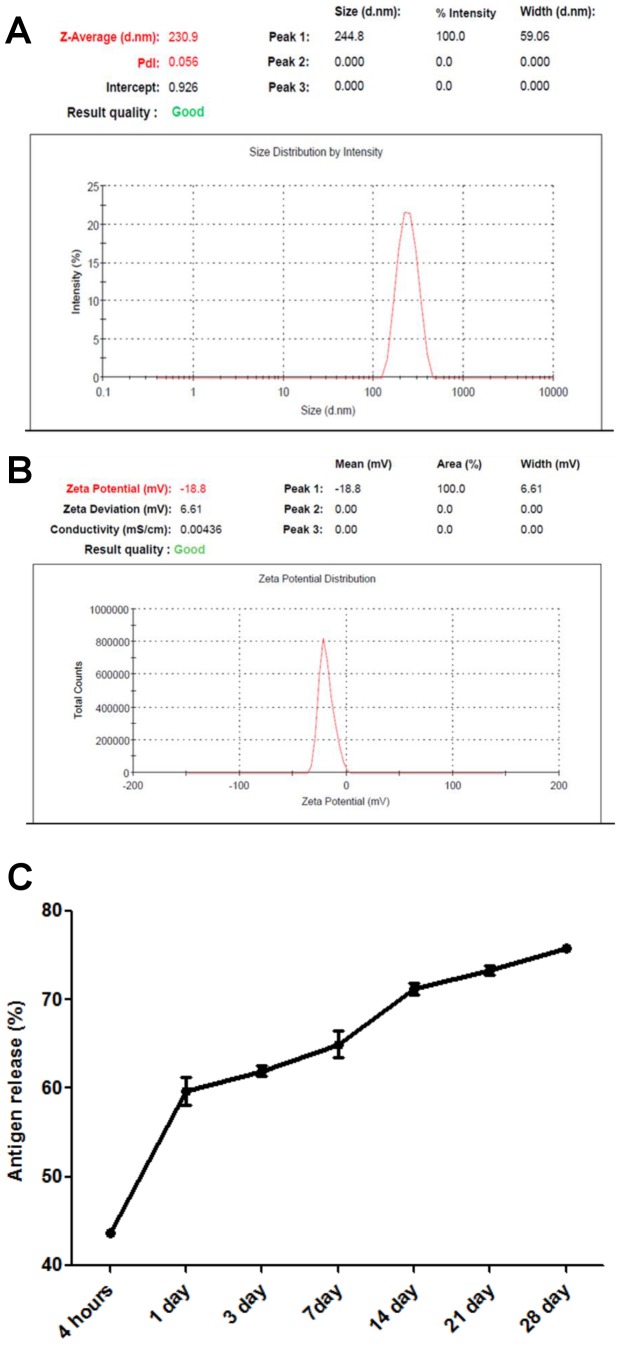
Physical characterization of PAD4-NP. A diluted preparation of PAD4-NP (0.1 mg/ml) was used for the estimation of particle size (**A**) and zeta potential (**B**). The z-average (d. nm) of PAD4-NP nanoparticles was 230.9 nm with peak at 244.8 nm and width of 59.06 nm. The nanoparticle formulation had polydispersity index of 0.056 which indicated the formulation to be monodisperse and devoid of particle aggregates. The zeta potential of PAD4-NP was −18.8 mV with 100% intensity at peak value −18.8 mV and width 6.61. Blank-NP with similar characteristics were used in the current study (refer text for details). (**C**) The *in vitro* release profile of PAD4 from PAD4-NP. The PAD4-NP suspended in PBS (50 mg/mL) were incubated at 37°C for indicated time periods and the release of PAD4 was estimated by micro-BCA assay. Experiment was done in triplicate. Note the initial burst release that caused >50% of PAD4 to get released within a day, was followed by a slower release kinetics that reached upto ∼75% of PAD4 release by day 28.

The immune responses generated by nanoformulations have been shown to be dependent on the size of nanoparticles comprising the formulation [17], [18], [30]. As we wanted to generate a strong humoral as well as cellular immune response, we optimized the nanoparticles preparation protocol to have nanoparticles smaller than 500 nm and preferentially in the size range of ∼200–250 nm as described previously [17], [30], [31] **.** The size of PAD4-NP made was estimated using dynamic light scattering (Fig. 3A). The size measurement did show that the z-average for nanoparticles preparation was 230.9 nm (d. nm). Furthermore, the DLS plot showed that the nanoparticles in the formulation were part of a single population (100% intensity) – a prerequisite for correlating the immunological response with the size of nanoparticles, with peak at 244.8 nm and width of 59.06 nm. The polydispersity index of nanoparticle formulation was 0.056 which indicated the formulation to be monodisperse and devoid of particle aggregates.

The zeta potential of PAD4-NP was −18.8 mV (Fig. 3B). The zeta potential of PAD4-NP also showed a single population of nanoparticles with 100% intensity at peak value −18.8 mV and width 6.61. The above characteristics of the PAD4-NP were considered good for evaluating the protective efficacy in mice models. Similarly, the z-average of blank nanoparticle (Blank-NP) used in current study was 243.5 nm. The polydispersity index and zeta potential of Blank-NP were 0.190 and −17.6 mV, respectively.

The *in vitro* controlled release kinetics study of PAD4-NP formulation (Fig. 3C) indicated that the release of PAD4 from PAD4-NP initially showed a burst release with ∼50% of PAD4 getting released within first 24 h, followed by a slow release kinetics so that ∼75% of PAD4 was released by 4 weeks.

### The PAD4-NP Elicited a High PAD4 Specific IgG Antibody Titer with Mixed IgG1/IgG2a Subtype Response

**Figure 4:**
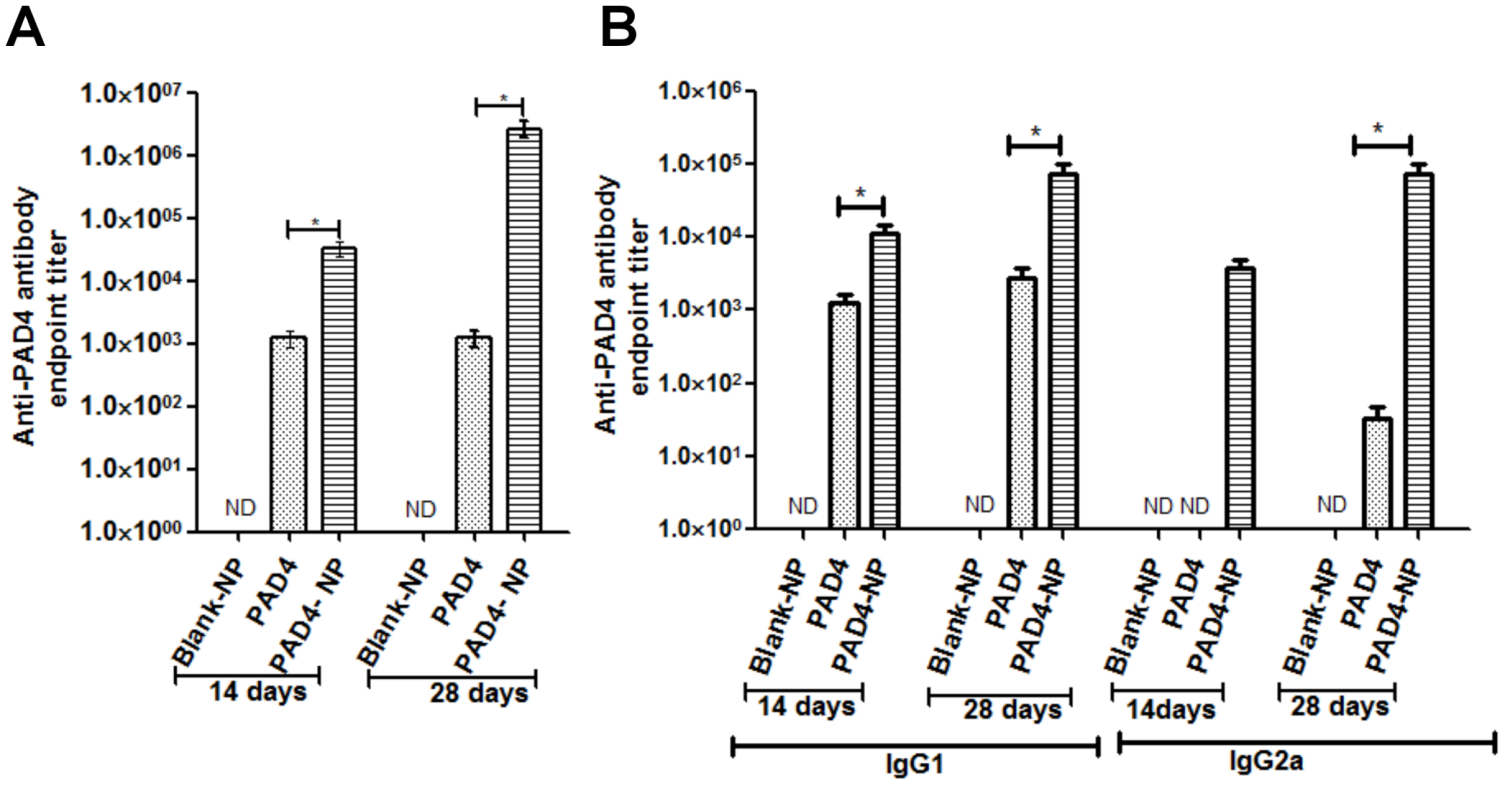
PAD4-NP elicits an enhanced immune response. Swiss Webster outbred mice were immunized with a single-dose of PAD4 or PAD4-NP (n = 8). The sera from immunized mice were collected on day 14 and day 28 post immunization. Serial dilutions of sera from each group were analyzed for IgG titer and IgG subtype titer using PAD4 specific ELISA. (**A**) The PAD4-NP induced high level of IgG titer that increased by more than 80 fold from day 14 to day 28 post immunization while PAD4 immunized mice did not show any increase in IgG titer from day 14 to day 28. (**B**) The PAD4-NP elicited both the IgG1 and IgG2a immune response while PAD4 elicited predominantly IgG1 response. The PAD4-NP produced significantly robust response as compared to PAD4. Note the antibody titers (Y axis) are presented on log scale. Error bars indicate ± SE of three experiments. The asterisk (*) denotes statistically significant change (P<0.05) between PAD4 and PAD4-NP groups.

The immune correlates of the single-dose and adjuvant-free PAD4-NP was compared with that of PAD4, PBS and Blank-NP in Swiss Webster outbred mice. The dose of PAD4 (50 µg/mice) used for the immunization, was based on a previous report [32]. A single dose of PAD4-NP encapsulating 100 µg of PAD4 was used for immunization as the initial release of ∼50% of PAD4 from PAD4-NP within a day (see Fig. 3C) could mimic adjuvant-free dose of PAD4, while its slow release later would show depot effect. The immune response assessment by PAD4 specific ELISA indicated that the single dose of PAD4-NP elicited the geometric mean IgG antibody titer of 30328 on day 14 that increased to 2457000 on day 28, whereas mice immunized with PAD4 did not show such enhancement in antibody titer from day 14 to day 28 and remained 1132 at both day 14 and 28 post immunization (Fig. 4A). This result indicated that the PAD4-NPs were capable of eliciting high titer of PAD4 specific IgG. The enhancement in the antibody titer from day 14 to day 28 with a single-dose immunization of PAD4-NP indicated that this formulation and vaccine schedule could elicit a classical immune response without the need of classical vaccination schedule, *i.e.,* multiple booster doses or repeated exposure to antigens. Furthermore, PAD4-NP generated the high antibody titer response without the help of any adjuvant. Blank-NP and PBS immunization did not elicit significant antibody titers.

The differential IgG subtype response is a major immune correlate in *Bacillus anthracis* infection [33], [34]. We evaluated the IgG1 and IgG2a subtype response in different mice groups (Fig. 4B). The immunization with PAD4-NP elicited the mixed IgG1 and IgG2a response, whereas PAD4 induced predominantly IgG1 immune response. The heterogeneous elicitation of IgG1 and IgG2a was similar to the immune response generated by whole PA and AVA [33], [34]. The mixed immune response was presumed to be conferring protective immunity against *Bacillus anthracis* spore challenge [33], [34].

### PAD4-NP Elicited a Mixed Th1/Th2 Cytokines

As a mixed antibody IgG1 and IgG2a subtype response was observed in PAD4-NP immunized mice, we explored whether this response was generated due to induced Th1 and Th2 heterogeneity [33]. We isolated the spleens from immunized outbred mice on the spore challenge day, *i.e.*, 40 days after the single-dose immunization. The splenocytes were stimulated *in vitro* with PAD4 or only medium (control group) and culture supernatants were collected after 36 h.

**Figure 5:**
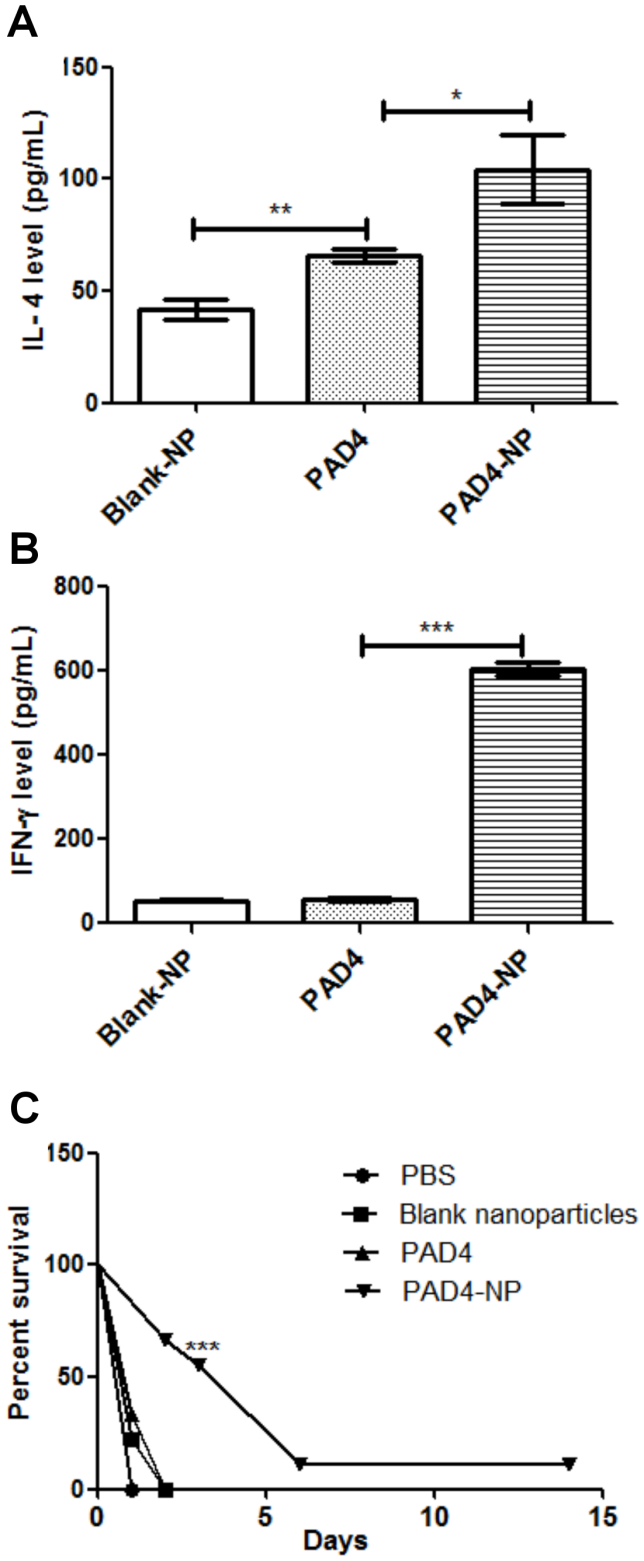
PAD4-NP elicits a robust heterogeneous Th1/Th2 response and protects outbred Swiss Webster mice against anthrax spore challenge. Splenocytes isolated on day 40 post immunization were stimulated *in vitro* with PAD4 or culture medium. PAD4-NP immunized mice elicited higher levels of IL-4 (**A**) and INF-gamma (**B**) as compared to that by PAD4 alone or Blank-NP. The cytokines levels (IL-4 and IFN-gamma) were estimated using opt-EIA kit (BD Bioscience Pharmingen) as per manufacturer’s instructions. Error bars in (A) and (B) indicate ± SE of three experiments done in triplicate. The statistical significance is highlighted (* for P value 0.01 to 0.05, ** for P values 0.01 to 0.001, *** for P values <0.001). (C) PAD4-NP enhanced the median survival in Swiss Webster outbred mice (n = 8) when challenged with anthrax spores (0.4 X10^8^ spores of *Bacillus anthracis* Sterne strain per mice). PAD4-NP immunized mice showed the median survival of 6 days while PAD4, PBS or Blank-NP immunized mice had median survival of 1 day.

PAD4-NP elicited a mixed Th1/Th2 response (Fig. 5A–B). The IFN-gamma level was 606.7±25.66 pg/mL, while IL4 level was 104.3±26.35 pg/mL in PAD4-NP immunized mice. The PAD4 immunized mice showed the IFN-gamma level of 56.67±7.638 pg/mL and IL4 level of 66±5.292 pg/mL, similar to control. These results indicated that the PAD4-NP elicited a mixed Th1/Th2 response [33].

### PAD4-NP Enhanced the Median Survival in Outbred Swiss Webster Mice

We evaluated the protective efficacy of PAD4-NP against *Bacillus anthracis* spore challenge in Swiss Webster outbred mice. The outbred mice model was selected for the challenge study as it mimics the real world scenario of MHC heterogeneity. The route of challenge was kept intraperitoneal as this route is known to stimulate the germination of anthrax spore, multiplication and further systemic invasion, thus reduces the interference of host innate resistance to *Bacillus anthracis* spore challenge [35]. The protective efficacy was evaluated in terms of median survival.

All mice groups (each group n  = 8), *i.e.*, mice immunized with single dose of PAD4-NP without any adjuvant, mice immunized with a single dose of PAD4 without any adjuvant, mice immunized with Blank-NP, and mice immunized with PBS, were challenged with 0.4×10^8^ spores/mice (Fig. 5C). As anthrax infection follows a distal mechanism of pathogenesis [36] and *Bacillus anthracis* observe a bottleneck in dissemination to distal organs [37], a high challenge dose was used to reduce such an interference from host innate resistance and generate a more systemic infection.

PAD4-NP immunized mice showed the median survival of 6 days with 11% survival, whereas PAD4 immunized mice had the median survival of 1 day (Fig. 5C). The PBS only and Blank-NP also had the median survival of 1day. All mice were observed up to 15 days post spore challenge. These results demonstrated the ability of PAD4-NP in eliciting a protective immune response following a single-dose and adjuvant-free vaccine schedule.

## Discussion

There have been a lot of efforts towards generation of better vaccines against anthrax that may be more effective, free of adjuvants and do not require booster doses. Multiple strategies have been explored including transgenic plant based vaccines [24], analogue of recombinant PA [38], PLGA-dendron nanoparticle based DNA vaccine of PA [39], rPA powder formulation [13]. Nonetheless, so far these efforts have not been very successful. Currently available vaccines, such as AVA, still have issues of adjuvant side effects, efficacy and booster dose requirement. Recently, there has been increased interest in delivering the vaccine candidates encapsulated in biodegradable and biocompatible polymer matrices (PLGA, polyamino acid, polysaccharides, *etc.*) to eliminate the problems associated with adjuvants, and circumvent the requirement of booster doses that remain the major challenge in effective implementation of any vaccination program.

In the present study, for the first time, we explored the possibility of encapsulating a recombinant antigen in PLGA nanoparticles as a single-dose and adjuvant-free formulation to generate immunity against anthrax. However, previously a polylactide (PLA) encapsulated recombinant PA microspheres containing candidate vaccine, formulated by w/o/w solvent evaporation method, was evaluated for eliciting protective immunity [21]. The encapsulated PA was found to induce anti-PA IgG titer of 79.17 and 555.57, whereas free PA induced the titer of 169.31 and 1044.79 on day 45 and 84, respectively [21]. This decrease in anti-PA IgG response after PLA encapsulation may reflect the loss of antigenic repertoire during encapsulation process due to the exposure of PA to the organic interface, sheer force or just exposure to low pH conditions during antigen release. It was reported previously that PA is structurally labile in acidic conditions [13]. In contrast to what was observed with PA encapsulation (*i.e.,* attenuated antigenicity/IgG response), PAD4 encapsulation elicited higher IgG response (*i.e.*, PAD4 nonencapsulated vs. PAD4-NP). This observed dichotomy, could be attributed to inherent instability of PA in various pharmaceutical preparation [13], [38], [40], [41] as opposed to PAD4 which was shown to withstand the harsh conditions such as low pH and still maintain the structural integrity to bind with anthrax toxin binding cell-receptors [22], and generate protective immune response [23], [24]. The crystal structure of PAD4 shows that it has the conformation similar to that of domain 4 in native PA molecule [22]. Interestingly, these properties of PAD4 cannot be harnessed for generating a robust protective immune response using whole PA molecule, as the PAD4 represents a labile domain when present in native PA molecule [28]. However, when we evaluated the PA encapsulated PLGA nanoparticles for its ability to generate immune response (unpublished data), our results indicated the immunogenicity of PA molecule in such formulation except no significant protection could be achieved as reported previously [21]. Combined, these observations suggest that the loss of critical epitopes of PA molecule, which could generate a protective immune response during the formulation procedure or later during antigen release at low pH due to PLGA degradation products, would be making PA unsuitable for such a vaccine formulation.

A soybean oil based nanoemulsion was previously explored for the formulation of PA based vaccine [5]. In that study, a biased Th1 response was observed with predominantly IgG2a response. The size of emulsion droplets prepared was less than 400 nm, comparable to the size of particles used in the present study. As they had used a novel nanoemulsion and the antigen release from it was neither explained in the cited literature nor the mechanism of antigen release from soybean oil emulsion is very well studied, it is difficult to explain the predominant Th1 response from their soybean oil based PA nanoemulsion vaccine. However, the antigen release mechanism from PLGA based nanoparticles have been extensively studied [17]–[19], [42]. PLGA nanoparticles undergo hydrolytic degradation via bulk erosion after the onset of initial burst release. The antigen release is important for shaping the immune response while the properties of polymeric material as such are not [43]. We observed the mixed Th1/Th2 response complimented by IgG1/IgG2a response in our studies involving PLGA-NP. This response can be attributed to the initial release of PAD4 antigen from PLGA-NP where free antigen could contribute to Th2 response, whereas targeted antigen could contribute to Th1 response. Similar to ours, PLGA nanoparticles have been shown to induce a heterogeneous Th1/Th2 response for *Plasmodium vivax* malaria vaccine [44] and leishmaniasis vaccine [45] as well. It has been also demonstrated that PLGA nanoparticle can even induce Th1 response for a Th2 biased peptide [46].

The importance of Fcγ receptors as the regulators of the protective immune response has been extensively established [47]. They are also known to play an important role in the antibody mediated bacterial toxin neutralization in anthrax [48]. It has been shown that IgG2a has the better neutralization potential than IgG1. As our PAD4-NP generated strong IgG2a response, it could be an important contributing factor in conferring a better protective immunity against anthrax spore challenge. It has been also reported that anti-PA IgG2a or Th1 response can initiate the complement fixation and antibody-dependent cellular toxicity which can be sporicidal or alternatively inhibit the spore germination [49], [50]. The IgG2 response was also documented as a useful immunological marker in a study that compared rPA/Ribi formulation with rPA/alhydrogel [15]. The rPA/Ribi formulation eliciting IgG2 response was shown to provide complete protection against anthrax spore challenge, whereas rPA/alhydrogel eliciting IgG1 response was shown to provide protection in upto 71% cases only [15].

In the present work, a simple methodology has been developed and employed to minimize or eliminate some of the most vexing problems associated with currently available anthrax vaccines. Although single-dose of adjuvant-free PLGA-NP elicited both Th1/Th2 response and generated PAD4 specific antibodies, the protection offered from spore challenge was not as good as obtained by other experimental vaccines [6] including ours which were based on recombinant full length PA or PAD4 [24]. The use of adjuvants and multiple boosters could be a few reasons for their better performance in comparison to PAD4-NP formulation. As elimination of adjuvants and booster dose requirement is desired to have better compliance, our work could be a first step in that right direction. Immediate future work may be focused on making PAD4-NP variants (e.g., size, surface characteristics for better mucosal adhesion and nasal immunization, inclusion of immune enhancers *etc.* [19]), and evaluate them for their ability to elicit protective immune response. The approach utilized here to encapsulate PAD4 was less technically challenging that can be further applied or replicated to combine other antigens or immune enhancers to develop a candidate single-dose adjuvant-free anthrax vaccine.
